# Medical and Physical Intelligence

**Published:** 1806-09

**Authors:** 


### MEDICAL AND PHYSICAL INTELLIGENCE.

Mr. John Shawe, surgeon, at Liverpool, has communicated to us a very effectual remedy for that species of inflamed eye, commonly called Egyptian ophthalmia. The following is an extract from his letter. "We had ophthalmia very prevalent here last summer; I believe it was very general throughout the country; try; the inflammation was unusually great, and extremely obstinate; the pain in the eyes almost insupportable, and particularly in attempting to open them, apparently as if sharp sand, or some rough substance was pricking of them; the tears felt very hot in trickling down the cheeks. Suddenly I became a sufferer, and for the first time in my life I had ever felt the pain of inflamed eyes. From the general description, it is one and the same called Egyptian ophthalmia. It occurred to me, that some mucilaginous body might relieve, by uniting with the tears and blunting their acrimony. Accordingly, I prepared some mucilage of quince seeds, and had it dropped into my eyes. The instant relief that followed this application exceeded my expectation, and far surpassed every means I had heard or tried before; the pricking sensation was eased almost instantly, and the eyes could be moved with freedom. This will suffice, without giving you the number, or any long history of others, who have received equal benefit. I have tried the mucilage of gum arabic and linseed, but without any benefit. Whether any peculiar property may be in the acid of the quince seeds uniting with the tears, and correcting their alkaline action, I do not presume to give my opinion, and shall leave that to the superior judgment of your Readers."

---

*Original Vaccine Pock Institution, Broad-street, Golden-square.*

At a Meeting, held on Tuesday, June 24, 1806; in consequence of adverse Reports, it was

RESOLVED,

1. That the Medical Establishment are of opinion that the Cow-pock Inoculation, under the regulations of this Institution, according to their experience, is decidedly advantageous over that of the Small-pox.

2. That since the offered reward of Five Guineas to any person who *has been certified*, not one has applied in consequence of a failure.

3. That in general, persons decline to accept the certificate, on account of their own experience shewing the security after vaccination, on living with patients ill of the Small-pox.

4. That as, in a small proportion, persons considered to have been duly vaccinated have taken the Small-pox, it is thought proper to grant certificates to those only who have been tested at this Institution.

5. To give further satisfaction, and considering that the vaccine disorder is a new morbid affection which requires investigation, (one of the great objects of this Institution) it is requested that any persons who apprehend they may have taken the Small-pox, or have any disorder from vaccination, will apply to the Board, any Tuesday or Friday, at one o'clock, to execute the design of a faithful publication of accumulated evidence.

---

The

The Autumnal Course of Lectures at St. Thomas's and Guy's Hospitals will commence in the following order:—

St. Thomas's Hospital.—Anatomy, and operations of surgery, by Mr. Cline and Mr. Cooper, Wednesday, October 1, at half past one.—Principles and Practice of Surgery, by Mr. Cooper, on Monday, October 6, at eight in the evening.

Guy's Hospital.—Practice of Medicine, by Dr. Babington and Dr. Curry, on Wednesday, October 1, at ten in the morning. Principles and Practice of Chemistry, by Dr. Babington and Mr. Allen, on Thursday, October 2, at ten in the morning.—Theory of Medicine, comprehending Pathology, Therapeutics, and *Materia Medica*, by Dr. Curry, on Friday, October 3, at eight in the evening.—Physiology, or Laws of the Animal Economy, by Dr. Haighton, on Monday, October 6, at a quarter before seven in the evening.—Midwifery, and Diseases of Women and Children, by Dr. Haighton, on Monday, October 6, at eight in the morning.—Clinical Lectures on Select Medical Cases, by Dr. Babington, Dr. Curry, and Dr. Marcet, from November till May.—Besides these, during the winter, a course of Lectures on the Structure and Diseases of the Teeth, will be given by Mr. Fox, Surgeon-Dentist; and one on Veterinary Medicine, by Mr. Coleman, Professor at the Veterinary College. These several Lectures are so arranged, that none of them interfere with the others in the hours of attendance; and the whole is calculated to form a complete course of medical and chirurgical instruction. Terms and other particulars to be learnt from Mr. Stocker, apothecary to Guy's Hospital, who is also empowered to enter gentlemen to such of the Lectures as are given at Guy's. Dr. Babington and Dr. Curry, take this opportunity of informing those gentlemen who have attended their Lectures on the Practice of Medicine within the last four years, that their Syllabus on that subject is now finished, and ready for delivery. Such gentlemen, then, as have received a part of the work, will get the remainder by applying at the Hospital, either personally, or through their friends, and stating how much is wanting to render it complete.

The Autumnal Courses of Lectures delivered at the London Hospital will commence on the first of October.—Theory and Practice of Physic, by Dr. Cooke.—Chemistry, by Dr. Hamilton, and Dr. Yelloly.—*Materia Medica*, by Dr. Frampton.—Theory and Practice of Midwifery, by Dr. Dennison.—Clinical Observations on Surgical Cases, by Sir William Blizard and Mr. Thomas Blizard.—Anatomy, Physiology, and the Operations of Surgery, by Mr. Headington and Mr. Frampton.—Anatomical Demonstrations and Dissection, Principles of, by Mr. Armiger.—Surgery, by Mr. Headington. Further particulars may be known by applying to Mr. Price, apothecary, at the Hospital.

Medical Theatre, St. Bartholomew's Hospital.—The following Courses of Lectures will be delivered at this Theatre the the ensuing winter.—On the Theory and Practice of Medicine, by Dr. Roberts and Dr. Powell.—On Anatomy and Physiology, by Mr. Abernethy.—On the Theory and Practice of Surgery, by Mr. Abernethy.—On Comparative Anatomy and the Laws of Organic Existence, by Mr. Macartney.—On Chemistry, by Dr. Edwards. —On Midwifery and the Diseases of Women and Children, by Dr. Thynne.—The Anatomical Demonstrations and Practical Anatomy, by Mr. Lawrence.—The Anatomical Lectures will begin on Wednesday, October 1, at two o'clock, and the other Lectures in the course of the same week.—Further particulars may be known by applying to Mr. Nicholson, at the Apothecary's shop, St. Bartholomew's Hospital.

UNIVERSITY OF GLASGOW.

The Medical Lectures in the University of Glasgow will begin on Tuesday the 4th of November, at the following hours:

Dietetics, Materia Medica, and Pharmacy, by Dr. MILLAR, at ten o'clock, forenoon.

Midwifery, by Mr. TOWERS, at eleven.

Theory and Practice of Physic, by Dr. FREER, at twelve.

Anatomy and Surgery, by Dr. JEFFRAY, at two o'clock, afternoon.

Chemistry and Chemical Pharmacy, by Dr. CLEGHORN, at seven.

Clinical Lectures on the Cases of Patients in the Royal Infirmary, on Thursday evening, the 13th of November, at six o'clock. The first Lecture by Dr. FREER.

Dr. BROWN will begin his Lectures on Botany, about the beginning of May next.

*Theatre of Anatomy, Great Windmill-Street.*

Mr. WILSON will begin the winter course of his Lectures on Anatomy, Physiology, Pathology, and Surgery, on Wednesday, Oct. the 1st, at two o'clock, as usual. A room will likewise be opened for Dissections, from nine in the morning till two in the afternoon, from the 10th of October till the 20th of April; where regular and full demonstrations of the parts dissected will be given, where the different cases in surgery will be explained, the methods of operating shewn on the dead body; and where also the various arts of injecting and making preparations will be taught.—The plan and terms of the course may be had in Great Windmill-street.

Mr. BROOKES will commence his Autumnal Course of Lectures on Anatomy, Physiology, and Surgery, on Wednesday, the 1st of October, at two o'clock, at his Theatre, Blenheim Street, Great Marlborough Street. The Dissecting Rooms will be open from eight o'clock in the morning till two, where Mr. Brookes attends.

Dr. BADHAM'S Lectures on Medicine and Chemistry will be commenced on Friday, 10th of October, at eight o'clock, at No. 15, Clifford-street, Burlington Gardens.

Dr. BRADLEY will commence his Autumnal Curse of Lectures on the Theory and Practice of Medicine, on Monday the 13th of October next, at Whitehall.

Dr,

Mr. A. CARLISLE, F. R. S. F. L. S. and Surgeon to the Westminster Hospital, intends to deliver a Course of Lectures on the Art and Practice of Surgery, in all its branches, during the present season, at his house in Soho-square.

An Introductory Discourse, (open to all Students) comprising the plan of these Lectures, will be given on Monday, the 13th of October, 1806, at seven o'clock in the evening. And the lectures will be continued on Mondays, Wednesdays, and Fridays, at the same hour.

Dr. CLARKE and Mr. CLARKE will begin their winter course of Lectures on Midwifery, and the Diseases of Women and Children, on Monday, October the 6th, at the Lecture Room, No. 10, Upper John-street, Golden-square. The lectures are read every day, (Sundays excepted) from a quarter past ten o'clock in the morning till a quarter past eleven, for the convenience of Students attending the Hospitals.—For farther particulars apply to Dr. CLARKE, No. 1, New Burlington-street; or to Mr. CLARKE, at his house, No. 10, Upper John-street, Golden-square.

In the first week of October, will commence a Course of Lectures on the Practice of Physic, Therapeutics, and Chemistry, in the Lecture-room, No. 9, Great George-street, Hanover-square, (removed from Leicester-square,) at the usual morning hours, viz. the medical lectures at eight, and the chemical at a quarter after nine o'clock; by George Pearson, M. D. F.R.S. senior physician of St. George's Hospital, of the College of Physicians, &c. A register is kept of Dr. Pearson's Cases in St. George's Hospital, and an account is given of them every Saturday morning, at a Clinical Lecture, at nine o'clock.

Dr. REID's Winter Course of Lectures on the Theory and Practice of Medicine, will commence on the 20th of October next, at his house, No. 6, Grenville Street, Brunswick Square, where Particulars may be had.

Mr. HOME's Lectures on the principal Operations of Surgery, given gratuitously to the Pupils of St. George's Hospital, will commence in October next as usual.—Mr. Gunning will likewise give Lectures on the Lues Venerea. &c.

Mr. GUNNING, Surgeon Extraordinary to his Royal Highness the Duke of Sussex, and Surgeon to St. George's Hospital, will commence his Course of Lectures on the Principles and Practice of Surgery, at his house, No. 43, Conduit-street, Hanover-square, on Monday, October 13, at seven o'clock in the evening. Further particulars may be known by applying to Mr. Gunning, in Conduit-street, or at St. George's Hospital.

Mr. THOMAS's Autumnal Course of Lectures, on the Theory and Principles of Surgery, will commence, as usual, early in October. Particulars may be known at his house, Leicester Place, or at the Anatomical Theatre, Windmill-street.

Mr.

Mr. Moor, Surgeon Dentist to her Royal Highness the Duchess of York, will commence a course of Lectures on the Structure and Diseases of the Teeth, some time in November, in which will be explained the complete practice of the Dentist.

Mr. Thelwall's Course of Evening Lectures, on the Physiology of Elocution, or those parts of the animal œconomy that are connected with the phenomena of Speech, and on the nature, causes, and treatment of Impediments, will commence on Wednesday, October 1, at his house in Bedford Place. They will be preceded by a preparatory series of Literary Lectures, which will commence in the second week of September, and will be nine in number. It is the object of Mr. T's longer course, to treat in a popular way, every part of the extensive subject of Elocutionary Science, and Elocutionary Accomplishment:—the highest graces and excellences of which, as well as the simplest requisites of intelligible facility, he professes to trace from fundamental principles of physical and musical science. The Scientific Lectures will be delivered every Wednesday and Friday; the critical and oratorical portions of the subject, every Monday evening. For the accommodation of such students, as are desirous of taking up the subject in a more profound and abstract point of view, morning Lectures are also intended to be delivered to select classes :- and as there are parts of the subject that may be expected to be particularly interesting to medical students, arrangements will be made to interfere as little as possible with other branches of professional study. Mr. T. continues to give private instructions to pupils, who are afflicted with impediments of enunciation, or with feebleness, dissonance, or other defects of voice.

The Society for bettering the condition of the poor, have the satisfaction to find that contagious fever has scarcely made its appearance in the metropolis during the last two years.

Dr. Adams's second edition of his work on Morbid Poisons is nearly printed off.

---

